# Incremental Predictive Value of Conventional MRI Pelvimetry for Major Low Anterior Resection Syndrome After Rectal Cancer Surgery

**DOI:** 10.3390/jcm15187145

**Published:** 2026-09-15

**Authors:** Stefan Morarasu, Gabriel Mihail Dimofte, Constantin Osman, Wee Liam Ong, Tudor Gramada, Sorinel Lunca

**Affiliations:** 1 Grigore T. Popa University of Medicine and Pharmacy, 700115 Iasi, Romania; morarasu.stefan@gmail.com (S.M.); osmanconstantin94@gmail.com (C.O.); william05021990@gmail.com (W.L.O.); tudorgramada@gmail.com (T.G.); sdlunca@yahoo.com (S.L.); 2 2nd Department of Surgical Oncology, Regional Institute of Oncology, 700483 Iasi, Romania; 3 Department of Radiology, Regional Institute of Oncology, 700483 Iasi, Romania

**Keywords:** rectal cancer, low anterior resection syndrome, LARS, MRI pelvimetry, skeletal pelvimetry, functional outcomes, prediction model

## Abstract

**Background**: Low anterior resection syndrome (LARS) is a frequent functional complication after sphincter-preserving rectal cancer surgery. Although tumor location and treatment-related factors are established determinants, the contribution of pelvic skeletal anatomy assessed by magnetic resonance imaging (MRI) pelvimetry remains uncertain. This study evaluated whether three conventional MRI-derived skeletal pelvimetry measurements provide incremental predictive information for major LARS beyond a parsimonious preoperative clinical reference model. **Methods**: This single-center observational cohort study included 168 patients who underwent sphincter-preserving rectal cancer surgery and completed postoperative LARS assessment. MRI pelvimetry included anteroposterior pelvic diameter, interspinous distance, and sacral curvature depth. Associations with LARS score and major LARS were evaluated using correlation analyses, pelvic-tertile comparisons, and multivariable logistic regression. Firth penalized logistic regression was prespecified as a sensitivity analysis. The primary clinical reference model included age, categorical tumor position, and neoadjuvant radiotherapy. Internal validation used stratified five-fold cross-validation repeated 100 times. Incremental model fit was assessed using a likelihood-ratio test. **Results**: Major LARS occurred in 48 patients (28.6%), and the median postoperative LARS score was 18 (interquartile range 9–31). LARS scores and major LARS prevalence were similar across anteroposterior pelvic diameter tertiles. In the parsimonious multivariable model, low tumor position was associated with major LARS (OR 9.51, 95% CI 2.00–45.15; *p* = 0.005), whereas anteroposterior pelvic diameter and interspinous distance were not associated with major LARS. The parsimonious preoperative clinical model achieved a mean repeated five-fold cross-validated AUC of 0.669, compared with 0.671 after addition of MRI pelvimetry (ΔAUC = +0.002, 95% CI −0.052 to 0.054), with no statistically detectable improvement in nested-model fit (likelihood-ratio *p* = 0.122). Brier scores were 0.187 for both models. Calibration, overall prediction error, and decision-curve performance were similar between models. **Conclusions**: In this selected retrospective cohort, the three conventional MRI-derived skeletal pelvimetry measurements evaluated did not demonstrate a clear incremental improvement in prediction of major LARS beyond the parsimonious preoperative clinical reference model.

## 1. Introduction

Low anterior resection syndrome (LARS) represents one of the most prevalent and clinically significant long-term sequelae following sphincter-preserving rectal cancer surgery, affecting up to 50–60% of survivors and substantially impairing quality of life, social participation, and psychological well-being [1,2,3]. Rather than a single entity, LARS encompasses a multidimensional constellation of bowel dysfunction, including urgency, clustering, increased stool frequency, and fecal incontinence, reflecting complex pathophysiological interactions between neorectal reservoir dysfunction, altered anorectal sensation, pelvic floor neuromuscular injury, and autonomic denervation [4,5,6]. The clinical relevance of LARS has been reinforced by the widespread adoption of patient-reported outcome tools such as the LARS score and by international consensus definitions emphasizing patient-centered functional consequences [7,8,9].

Despite advances in surgical technique, minimally invasive approaches, and multimodal oncologic therapy, functional outcomes after rectal cancer surgery remain heterogeneous. Classical determinants of LARS severity include low tumor height, total mesorectal excision (TME), neoadjuvant radiotherapy, and the use of diverting stomas [10,11,12]. Among these factors, pelvic radiotherapy has attracted particular attention because of its biological effects on anorectal tissues, including fibrosis, vascular injury, impaired compliance, and neuromuscular dysfunction of the sphincter complex [13,14,15]. However, radiotherapy alone does not fully explain inter-individual variability in functional outcomes, suggesting that host-specific anatomical and physiological factors may modulate susceptibility to postoperative dysfunction.

Pelvic anatomy represents a biologically plausible determinant of LARS. Anatomical constraints influence surgical exposure, nerve preservation, anastomotic configuration, and postoperative reservoir geometry, all of which may affect bowel function. Narrow pelvic dimensions, deeper sacral curvature, and reduced interspinous distance have been associated with increased surgical difficulty and oncologic outcomes such as circumferential margin status [16,17,18]. Magnetic resonance imaging (MRI), routinely used for rectal cancer staging, provides quantitative pelvimetry parameters without additional burden to patients, making it an attractive candidate for preoperative risk stratification [19,20,21]. Nevertheless, while MRI pelvimetry has been extensively studied in relation to operative complexity, its role in predicting postoperative functional outcomes has been far less explored.

Emerging evidence suggests that structural anatomical factors may influence continence mechanisms and bowel function after rectal surgery. Residual rectal length, tumor distance to the anal verge, pelvic floor morphology, and anorectal angle configuration have all been linked to functional outcomes [14,16,22]. Studies incorporating pelvic floor muscle measurements and imaging-based morphometric analyses further support the concept that anatomical variability contributes to postoperative bowel dysfunction [23,24]. However, direct evaluation of pelvic bony anatomy using MRI pelvimetry as an independent predictor of major LARS remains limited, with available studies characterized by small cohorts, heterogeneous methodologies, or inadequate adjustment for confounding surgical factors [17,18,19,20]. Consequently, it remains uncertain whether conventional skeletal measurements obtainable from routine preoperative MRI provide clinically meaningful predictive information for major LARS beyond established clinical factors.

The primary objective of this study was to determine whether three conventional MRI-derived skeletal pelvimetry measurements provide incremental predictive information for major LARS beyond a parsimonious preoperative clinical reference model. Secondary analyses evaluated the adjusted associations of individual pelvimetry measurements and selected clinical factors with major LARS.

## 2. Materials and Methods

### 2.1. Study Design and Setting

This retrospective, single-center observational cohort study included patients with rectal cancer treated at our institution between 2013 and 2024. Preoperative assessment included pelvic MRI, colonoscopy, and standard staging investigations. Decisions regarding neoadjuvant treatment, operative approach, and diverting stoma formation were made by a multidisciplinary tumor board. All oncologic treatment, surgery, and follow-up were performed at the same institution.

The present study represents a secondary imaging analysis of an institutional rectal cancer cohort previously reported in a study of factors associated with postoperative LARS [25]. The previous study included 182 patients, of whom 168 had complete measurements for all three MRI pelvimetry parameters and constituted the present analytic cohort. Thus, the current cohort is entirely contained within the previously reported population, and the postoperative LARS outcome data themselves are not novel. The distinct objective of the present analysis was to link these functional outcomes to newly performed standardized MRI pelvimetry measurements and to evaluate whether the three conventional skeletal measurements provide incremental predictive information for major LARS beyond preoperatively available clinical factors. Pelvimetry measurements were performed after completion of all postoperative LARS telephone assessments, with readers blinded to functional outcomes.

Postoperative bowel function was assessed using a Romanian-language translation of the standard five-item LARS questionnaire, based on the original instrument developed by Emmertsen and Laurberg [8]. The Romanian-language version used in this cohort has not undergone formal linguistic or psychometric validation. The questionnaire assesses incontinence for flatus, incontinence for liquid stool, bowel frequency, clustering, and urgency, generating a total score from 0 to 42 and classifying patients as having no LARS (0–20), minor LARS (21–29), or major LARS (30–42). Before data collection, both interviewers received standardized training from senior colorectal consultants. They were instructed to administer the questions in their predefined order, use objective and plain language without leading explanations, and record responses according to the questionnaire’s fixed response categories, which served as the standardized interview script. Structured telephone interviews were conducted by two senior surgical trainees, with each patient interviewed once by one interviewer. The interviewers were not blinded to clinical and treatment-related information; however, they were blinded to MRI pelvimetry measurements because all functional interviews had been completed before pelvimetry was performed. The interval to LARS assessment was calculated from surgery in patients without a diverting ileostomy and from ileostomy closure in patients who underwent diversion, thereby using restoration of bowel continuity as the functional starting point in diverted patients. Total LARS score, severity category, individual symptoms, and use of symptom-directed treatment were recorded. Formal inter-interviewer agreement was not assessed because interviews were not duplicated or recorded. Standardized preoperative LARS assessments were not available for this cohort.

The study was reported in accordance with STROBE guidelines. Ethical review and approval were waived by our Institution’s Ethics Committee because the study was observational and non-interventional, posed no additional risk to patients, and the data used for analysis were anonymized. Verbal informed consent was obtained from each participant before administration of the questionnaire.

### 2.2. Eligibility Criteria

Patients were eligible if they underwent sphincter-preserving rectal resection with restoration of bowel continuity and completed postoperative LARS assessment.

Exclusion criteria were a persistent diverting stoma at functional assessment, inability to provide reliable responses, death before functional follow-up, non-response or refusal to participate, non-operative management including a watch-and-wait strategy, and associated colon resection.

### 2.3. Data Collection

Clinical, operative, oncologic, and radiological data were extracted from institutional medical records and the radiology database. Collected variables included age, sex, BMI, diabetes mellitus, smoking history, tumor position, MRI-measured distance from the anal verge, pelvic dimensions, neoadjuvant radiotherapy and chemotherapy. Collected surgical variables included operative approach, extent of mesorectal excision (total versus partial mesorectal excision), anastomotic configuration (end-to-end versus side-to-end), anastomotic technique (stapled versus hand-sewn), diverting ileostomy, and anastomotic leakage/pelvic sepsis.

**Figure 1:**
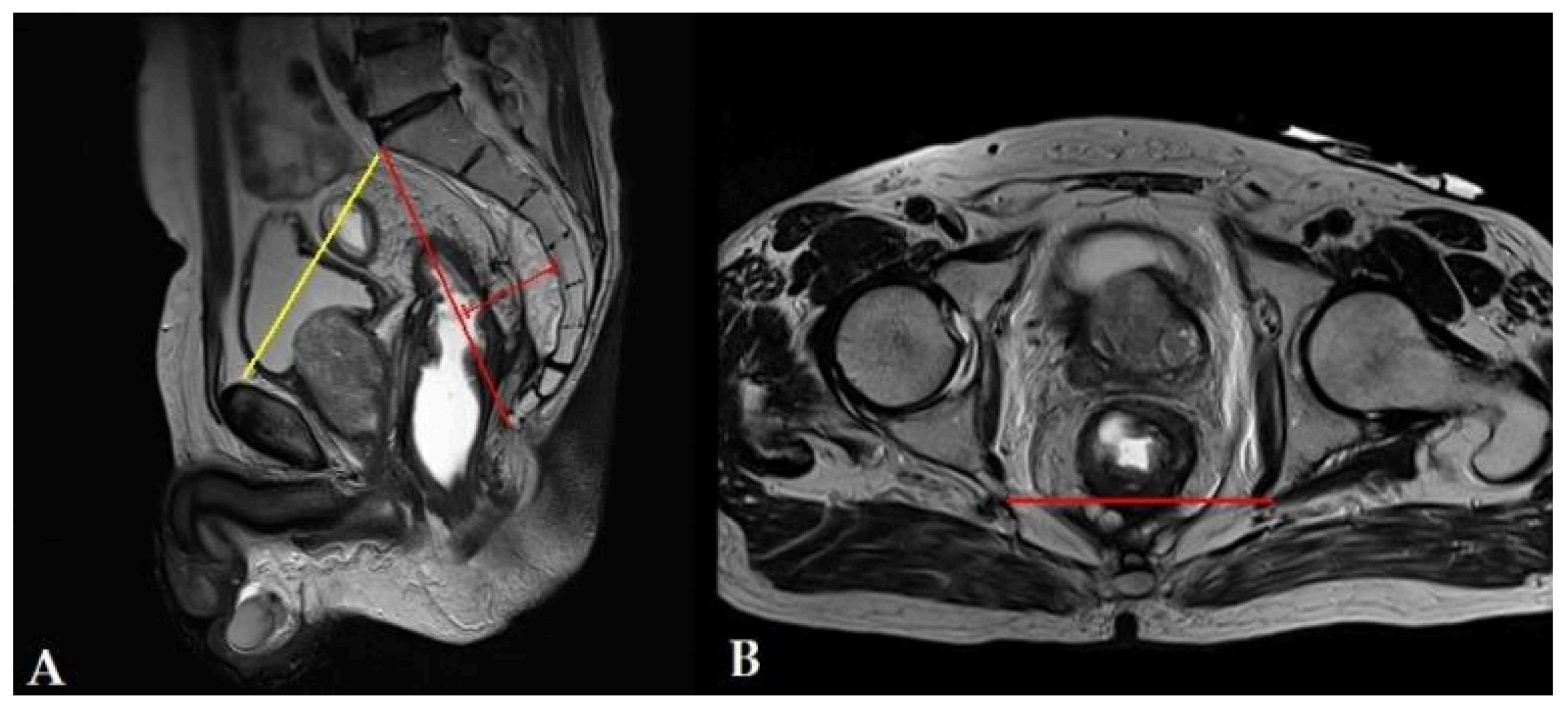
MRI landmarks used for conventional skeletal pelvimetry. (**A**) Midsagittal high-resolution T2-weighted image illustrating measurement of the AP pelvic diameter from the sacral promontory to the upper internal border of the pubic symphysis (yellow line) and sacral curvature depth as the maximum perpendicular distance from the promontory-to-coccyx reference line to the furthest anterior cortical surface of the sacrum (red lines). (**B**) Axial T2-weighted image illustrating interspinous distance measured between the tips of the right and left ischial spines (red line).

Tumor position was classified on preoperative MRI as low (<5 cm), middle (5–10 cm), or upper (>10 cm) according to the distance between the inferior tumor margin and the anal verge. Categorical tumor position was available for the entire analytical cohort from clinical and imaging records. Exact tumor distance from the anal verge was not systematically documented as a continuous numerical value and was available in 131 patients; consequently, continuous tumor distance was evaluated only as a sensitivity analysis in this subset. MRI pelvimetry was performed on high-resolution T2-weighted images, using multiplanar reconstructions. Measurements were obtained using RadiAnt DICOM Viewer version 4.2.1 (Medixant, Poznan, Poland) and its dedicated electronic ruler. Three conventional skeletal pelvimetry parameters were evaluated (Figure 1). Anteroposterior (AP) pelvic diameter was measured in the midsagittal plane from the most anterosuperior point of the sacral promontory to the upper posterior border of the pubic symphysis. Interspinous distance was measured in the axial plane between the tips of the right and left ischial spines. Sacral curvature depth was measured in the midsagittal plane by drawing a straight reference line from the sacral promontory to the tip of the coccyx and measuring the maximum perpendicular distance from this line to the furthest anterior cortical surface of the sacrum. The measurements used in the primary analyses were obtained by the primary reader, who was blinded to postoperative functional outcomes. To assess interobserver reproducibility, the same MRI examinations were independently measured by a second reader using the same anatomical definitions and measurement protocol. Interobserver agreement was quantified using a two-way random-effects, absolute-agreement, single-measurement intraclass correlation coefficient [ICC(2,1)] with 95% confidence intervals. Bland–Altman analysis was additionally performed to quantify mean interobserver differences and 95% limits of agreement.

The primary outcome was major LARS, analyzed against no or minor LARS. Secondary outcomes included total LARS score, three-category LARS severity, individual symptoms, assessment interval, and symptom-directed treatment.

### 2.4. Statistical Analysis

Continuous variables are reported as median and interquartile range, and categorical variables as number and percentage. Continuous variables were compared using the Mann–Whitney U test or Kruskal–Wallis test, as appropriate. Categorical variables were compared using the χ^2^ test or Fisher’s exact test. Associations between pelvimetry measurements and LARS score were assessed using Spearman correlation coefficients. Patients were stratified into tertiles according to anteroposterior pelvic diameter: narrow (≤108.0 mm), intermediate (>108.0 to ≤116.16 mm), and wide (>116.16 mm). LARS score and major LARS prevalence were compared across tertiles. Anteroposterior pelvic diameter was also evaluated as a continuous variable and modeled using restricted cubic splines to assess potential non-linearity in its association with major LARS. Average marginal predicted probabilities with bootstrap-derived 95% confidence intervals were plotted across the observed range. Because categorical tumor position and continuous tumor distance from the anal verge represent the same anatomical construct, categorical tumor position was used in the primary multivariable analyses. Continuous tumor distance was evaluated separately in patients with valid measurements.

### 2.5. Association and Prediction Analyses

Univariable logistic regression analyses were performed with major LARS as the dependent variable. Two complementary multivariable model specifications were used for distinct analytical purposes. First, a parsimonious association model included categorical tumor position, diverting ileostomy, and the three MRI pelvimetry variables: anteroposterior pelvic diameter, interspinous distance, and sacral curvature depth. This secondary analysis was used to estimate adjusted associations of individual anatomical and clinical factors with major LARS. For the primary evaluation of incremental predictive value, a parsimonious preoperative clinical reference model was prespecified, taking into account the limited number of major-LARS events (n = 48). The model included age, categorical tumor position, and neoadjuvant radiotherapy. These variables were selected on the basis of established or clinically plausible relationships with postoperative bowel dysfunction, availability before surgery, and the need to limit model complexity, rather than univariable statistical significance. Categorical tumor position was represented by two indicator variables, yielding four parameters in the clinical reference model. The corresponding clinical + pelvimetry model additionally included anteroposterior pelvic diameter, interspinous distance, and sacral curvature depth, yielding seven parameters in total. The intended prediction time point was after completion of preoperative staging and neoadjuvant treatment, when applicable, but before rectal resection; accordingly, all predictors in the primary prediction models were available before surgery. Standard maximum-likelihood logistic regression was used for the primary multivariable analyses. Firth penalized logistic regression using the predictors from the parsimonious effect-estimation model was prespecified as a sensitivity analysis. Adjusted odds ratios with 95% confidence intervals were reported. For clinical interpretability, effect estimates for continuous pelvimetry measurements were expressed per 10-mm increase. Because postoperative bowel function may vary according to the timing of assessment, an additional sensitivity model included the interval to LARS assessment. To further examine temporal heterogeneity, patients were categorized according to the interval to LARS assessment (<12, 12–24, 24–60, and ≥60 months), and the multivariable analysis was repeated in patients assessed ≥12 months after surgery or restoration of bowel continuity, as applicable.

The incremental contribution of MRI pelvimetry was assessed by likelihood-ratio comparison of the nested clinical and clinical + pelvimetry models. Predictive discrimination was assessed using the area under the receiver operating characteristic curve (AUC). Internal validation was performed using stratified five-fold cross-validation repeated 100 times, with approximately the proportion of major-LARS events preserved within each fold and identical resampling partitions used for the two models. Within each repetition, each model was fitted exclusively in four training folds and predicted probabilities were generated for patients in the held-out fold. This process was repeated across all five folds so that each patient received one out-of-fold prediction per repetition. The five held-out sets were recombined, an AUC was calculated from the complete out-of-fold prediction vector for each repetition, and discrimination was summarized as the mean AUC across the 100 repetitions. For estimation of uncertainty in incremental discrimination and assessment of calibration, the 100 out-of-fold predicted probabilities obtained for each patient were averaged to produce one internally validated predicted probability per patient. Incremental discrimination was quantified as the paired difference in AUC (ΔAUC) between the clinical + pelvimetry and clinical models, with a 95% confidence interval estimated using 10,000 paired patient-level bootstrap resamples. Calibration was assessed from these patient-level mean out-of-fold probabilities using the calibration intercept, calibration slope, Brier score, and graphical calibration plots. Clinical utility was evaluated using decision-curve analysis based on the patient-level mean repeated out-of-fold predicted probabilities over threshold probabilities from 0.10 to 0.50, comparing the net benefit of the two models with treat-all and treat-none strategies. Interobserver reproducibility of MRI pelvimetry measurements was assessed using two-way random-effects, absolute-agreement, single-measurement intraclass correlation coefficients [ICC(2,1)] with 95% confidence intervals and Bland–Altman analysis. Missing data were handled by complete-case analysis. All tests were two-sided, with *p* < 0.05 considered statistically significant. Statistical analyses were performed using MedCalc version 23.4.5 (MedCalc Software Ltd., Ostend, Belgium) and XLSTAT version 2024.3 (Addinsoft, Paris, France).

## 3. Results

### 3.1. Study Population and Overall Functional Outcomes

**Figure 2:**
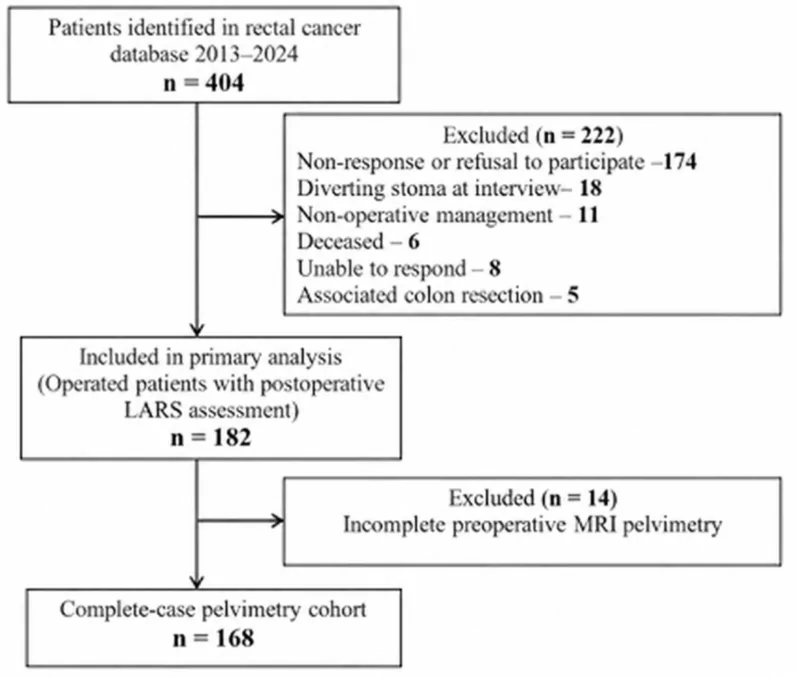
STROBE flowchart of patient selection.

A total of 404 patients with rectal cancer were screened. After application of the eligibility criteria, 168 patients who underwent sphincter-preserving surgery, completed postoperative LARS assessment, and had complete MRI pelvimetry constituted the final analytic cohort (Figure 2). Compared with patients excluded because of incomplete pelvimetry, included patients had similar age, sex distribution, BMI, and chemotherapy exposure, whereas tumor-position distribution and the use of neoadjuvant radiotherapy and diverting ileostomy differed between groups (Supplementary Table S1).

Major LARS occurred in 48 patients (28.6%), minor LARS in 25 (14.9%), and no LARS in 95 (56.5%). The median postoperative LARS score was 18 (IQR 9–31). Median scores were 10 (IQR 5–15.5) in patients without LARS, 26 (IQR 25–28) in those with minor LARS, and 36 (IQR 32–38.3) in those with major LARS (*p* < 0.001). Symptom-directed treatment was reported by 64 patients (38.1%), most commonly pharmacological treatment.

### 3.2. Baseline Characteristics of the Pelvimetry Cohort

Baseline demographic, clinical, anatomical, and surgical characteristics according to LARS severity are summarized in Table 1. Among the 131 patients with valid tumor-distance measurements, median distance from the anal verge was 88.5 mm (IQR 70–110) in patients without LARS, 87 mm (IQR 70–100) in those with minor LARS, and 82.5 mm (IQR 71.5–110) in those with major LARS (*p* = 0.815).

Age differed across LARS severity groups (*p* = 0.025), with patients with major LARS being younger. Tumor position, diverting ileostomy, neoadjuvant radiotherapy, and time to LARS assessment also differed across groups (*p* < 0.001, *p* = 0.007, *p* = 0.048, and *p* = 0.048, respectively). No significant differences were observed in sex, BMI, diabetes mellitus, smoking history, chemotherapy exposure, or the three MRI pelvimetry measurements. Surgical approach, extent of mesorectal excision, anastomotic configuration, and anastomotic leakage/pelvic sepsis did not differ significantly across LARS severity groups (Table 1).

### 3.3. Pelvic Anatomy and Functional Outcomes

**Figure 3:**
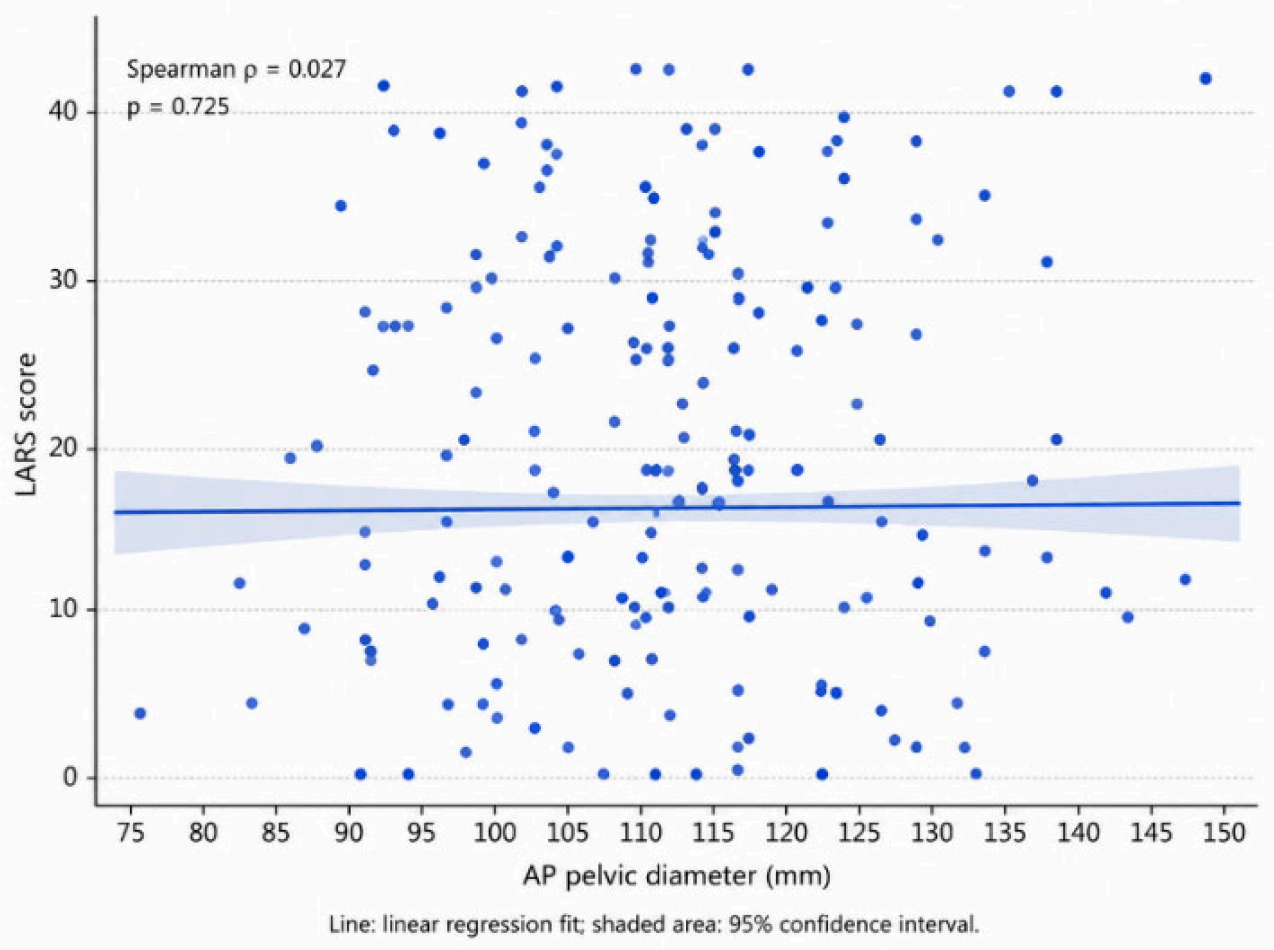
Postoperative LARS score according to anteroposterior pelvic diameter. Each point represents one patient. The fitted line and 95% confidence band are shown for visualization.

Anteroposterior pelvic diameter was not correlated with postoperative LARS score (Spearman ρ = 0.027, *p* = 0.725; Figure 3).

**Figure 4:**
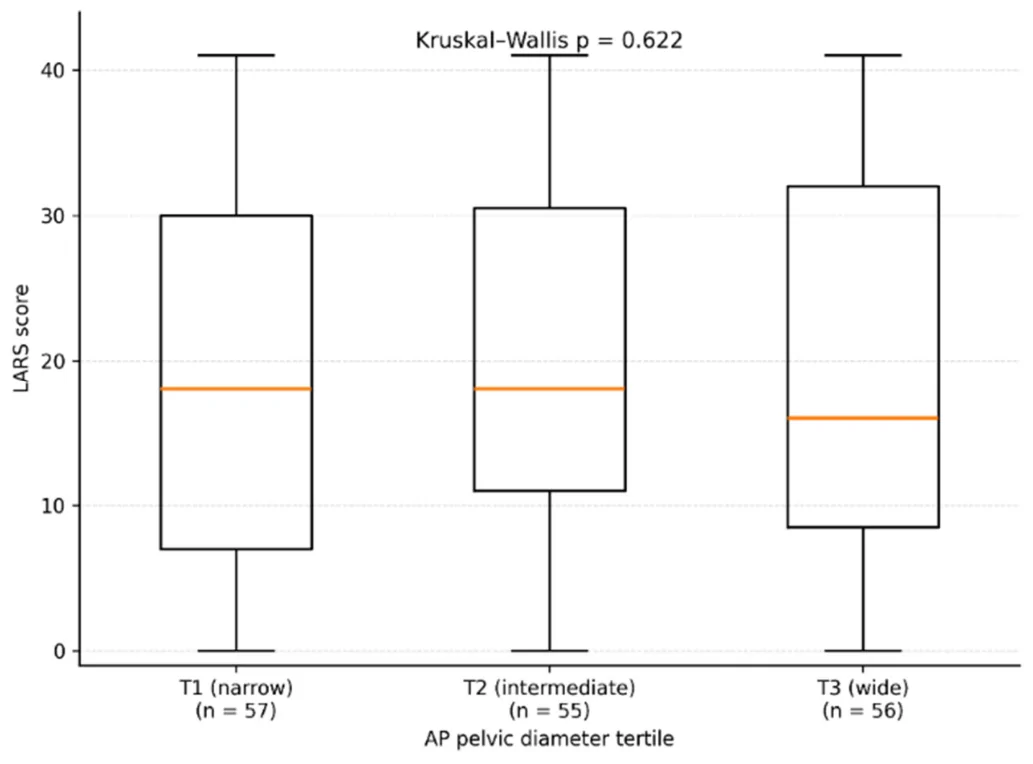
Distribution of postoperative LARS scores according to anteroposterior pelvic diameter tertile. Boxes represent the interquartile range, horizontal lines the median, and whiskers values within 1.5 times the interquartile range.

Median postoperative LARS scores were 18 (IQR 7–30) in the narrowest pelvic tertile, 18 (IQR 11–30.5) in the intermediate tertile, and 16 (IQR 8.5–32) in the widest tertile, with no difference across groups (*p* = 0.622; Table 2 and Figure 4).

**Figure 5:**
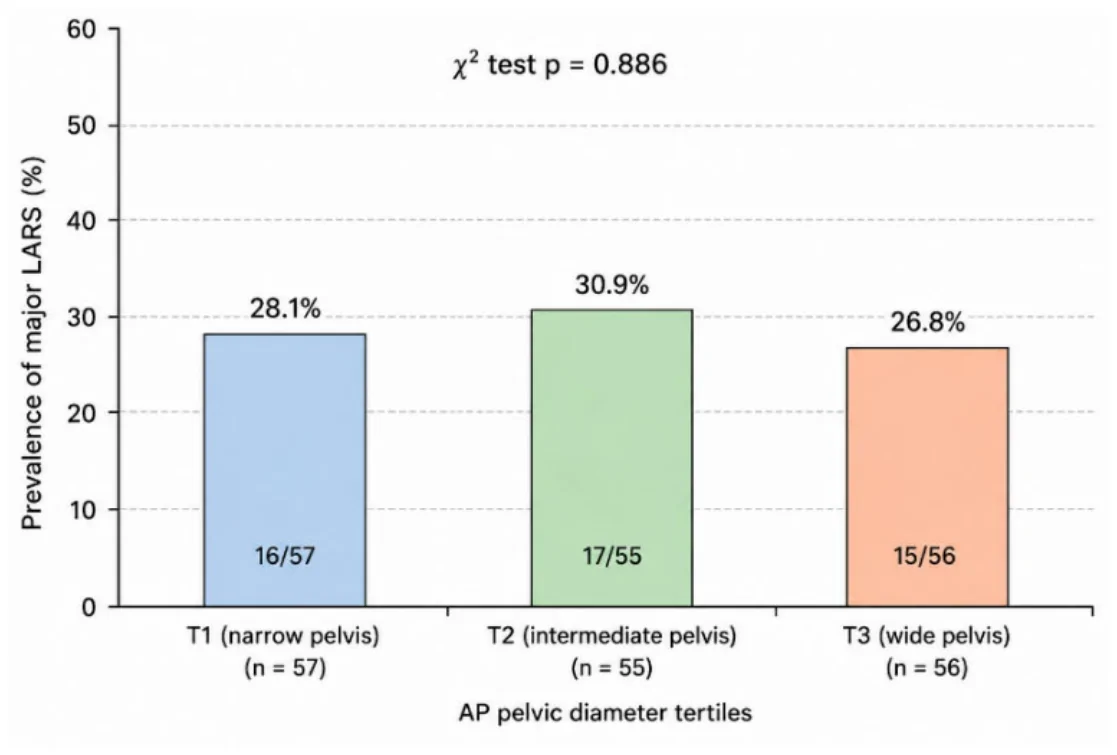
Prevalence of major LARS according to anteroposterior pelvic diameter tertiles.

Major LARS occurred in 28.1% of patients in the narrowest tertile, 30.9% in the intermediate tertile, and 26.8% in the widest tertile (*p* = 0.886; Table 2 and Figure 5).

**Figure 6:**
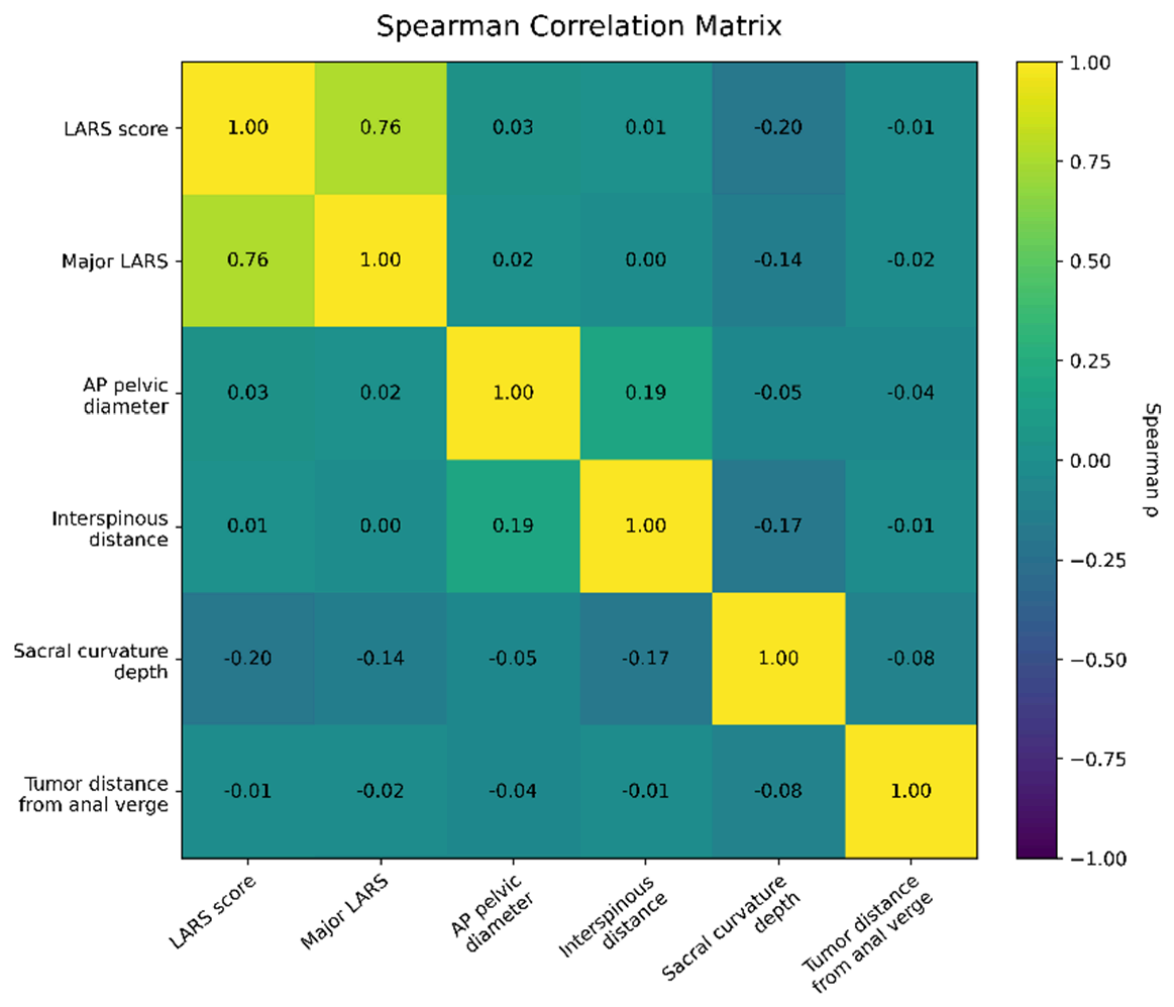
Spearman correlation matrix of MRI pelvimetry measurements, tumor distance from the anal verge, and postoperative LARS outcomes.

The three MRI pelvimetry measurements showed weak-to-moderate intercorrelations, whereas their correlations with postoperative LARS score were weak (Figure 6). An expanded correlation matrix is provided in Supplementary Table S2.

Interobserver reproducibility was excellent for all three MRI pelvimetry measurements. ICC(2,1) values were 0.992 (95% CI 0.990–0.994) for AP pelvic diameter, 0.990 (95% CI 0.988–0.992) for interspinous distance, and 0.985 (95% CI 0.981–0.988) for sacral curvature depth. Mean differences between the second and primary readers were +0.19, 0.00, and +0.11 mm, respectively. Bland–Altman 95% limits of agreement were −2.83 to +3.21 mm for AP pelvic diameter, −3.27 to +3.27 mm for interspinous distance, and −3.01 to +3.22 mm for sacral curvature depth (Supplementary Table S3).

### 3.4. Multivariable Analysis and Predictive Performance for Major LARS

In the secondary association analysis, a parsimonious multivariable logistic regression model evaluated the associations of tumor position, diverting ileostomy, and MRI pelvimetry parameters with major LARS (Table 3). For the primary prediction analysis, a parsimonious preoperative clinical reference model was used to evaluate the incremental predictive contribution of MRI pelvimetry (Table 4). Continuous tumor distance from the anal verge was evaluated separately in a sensitivity analysis.

In the parsimonious model, low tumor position was associated with higher odds of major LARS with a wide confidence interval (OR 9.51, 95% CI 2.00–45.15; *p* = 0.005). Middle tumor position was not associated with major LARS (OR 1.33, 95% CI 0.56–3.14; *p* = 0.513), whereas diverting ileostomy showed a borderline association (OR 2.58, 95% CI 0.98–6.77; *p* = 0.054). Anteroposterior pelvic diameter and interspinous distance were not associated with major LARS. Sacral curvature depth showed a small inverse association (OR 0.65 per 10 mm, 95% CI 0.44–0.98; *p* = 0.042).

Firth penalized logistic regression yielded similar estimates (Supplementary Table S4). Low tumor position remained associated with higher odds of major LARS, although with a wide confidence interval (penalized OR 7.96, 95% CI 1.97–38.95; *p* = 0.003). Middle tumor position, anteroposterior pelvic diameter (OR 1.04 per 10-mm increase, 95% CI 0.77–1.40; *p* = 0.803), and interspinous distance (OR 0.97 per 10-mm increase, 95% CI 0.70–1.34; *p* = 0.852) were not associated with major LARS. Diverting ileostomy showed an imprecise positive association (OR 2.44, 95% CI 0.99–6.48; *p* = 0.054), while sacral curvature depth showed an inverse association with considerable uncertainty (OR 0.67 per 10-mm increase, 95% CI 0.45–1.00; *p* = 0.052).

In a sensitivity analysis restricted to the 131 patients with valid tumor-distance measurements, continuous distance from the anal verge was not associated with major LARS. Median tumor distance was 82.5 mm (IQR 71.5–110.0) in patients with major LARS and 88.2 mm (IQR 70.0–110.0) in those with no/minor LARS (*p* = 0.815).

After adjustment for the interval between surgery (or restoration of bowel continuity) and LARS assessment, low tumor position remained associated with major LARS (OR 10.04, 95% CI 1.92–52.56; *p* = 0.006), whereas diverting ileostomy reached statistical significance (OR 2.73, 95% CI 1.02–7.34; *p* = 0.046). Longer follow-up was associated with lower odds of major LARS (OR 0.986 per month, 95% CI 0.974–0.998; *p* = 0.023). The remaining associations were unchanged (Supplementary Table S5).

The distribution of postoperative functional assessment also differed across follow-up intervals. Among 167 patients with valid assessment-time data, 28 were assessed <12 months, 38 at 12–24 months, 48 at 24–60 months, and 53 at ≥60 months. Major LARS occurred in 42.9%, 39.5%, 27.1%, and 15.1% of patients across these intervals, respectively (*p* = 0.021), while median LARS scores were 26, 19, 18, and 16, respectively (Supplementary Table S6 and Supplementary Figure S1).

When the analysis was restricted to the 139 patients assessed ≥12 months after surgery or restoration of bowel continuity, AP pelvic diameter (OR 1.11 per 10-mm increase, 95% CI 0.79–1.55; *p* = 0.561) and interspinous distance (OR 1.02, 95% CI 0.71–1.45; *p* = 0.927) remained unassociated with major LARS; the corresponding estimate for sacral curvature depth was OR 0.68 (95% CI 0.43–1.08; *p* = 0.101). Joint addition of the three pelvimetry measurements did not improve model fit (likelihood-ratio χ^2^ = 3.65, df = 3; *p* = 0.302; Supplementary Table S7).

**Figure 7:**
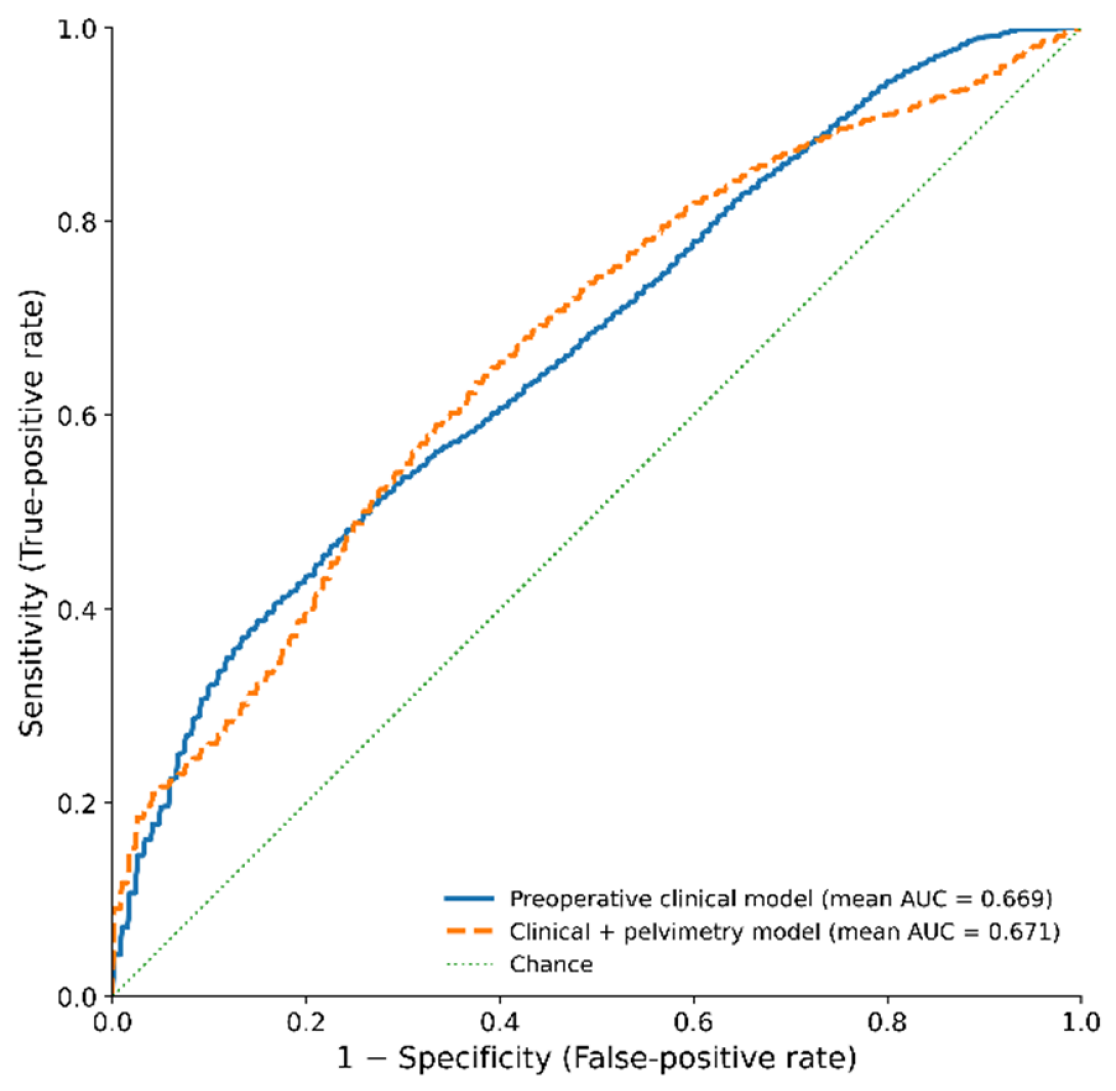
Repeated cross-validated receiver operating characteristic curves for prediction of major LARS. Curves represent mean out-of-fold ROC performance across 100 repetitions of stratified five-fold cross-validation for the parsimonious preoperative clinical model and clinical + pelvimetry model.

The parsimonious preoperative clinical model achieved a mean repeated five-fold cross-validated AUC of 0.669, compared with 0.671 for the clinical + pelvimetry model (ΔAUC = +0.002, 95% CI −0.052 to 0.054). Joint addition of the three pelvimetry variables did not produce a statistically detectable improvement in nested-model fit (likelihood-ratio χ^2^ = 5.79, df = 3; *p* = 0.122) (Figure 7 and Table 4, and Supplementary Table S9).

**Figure 8:**
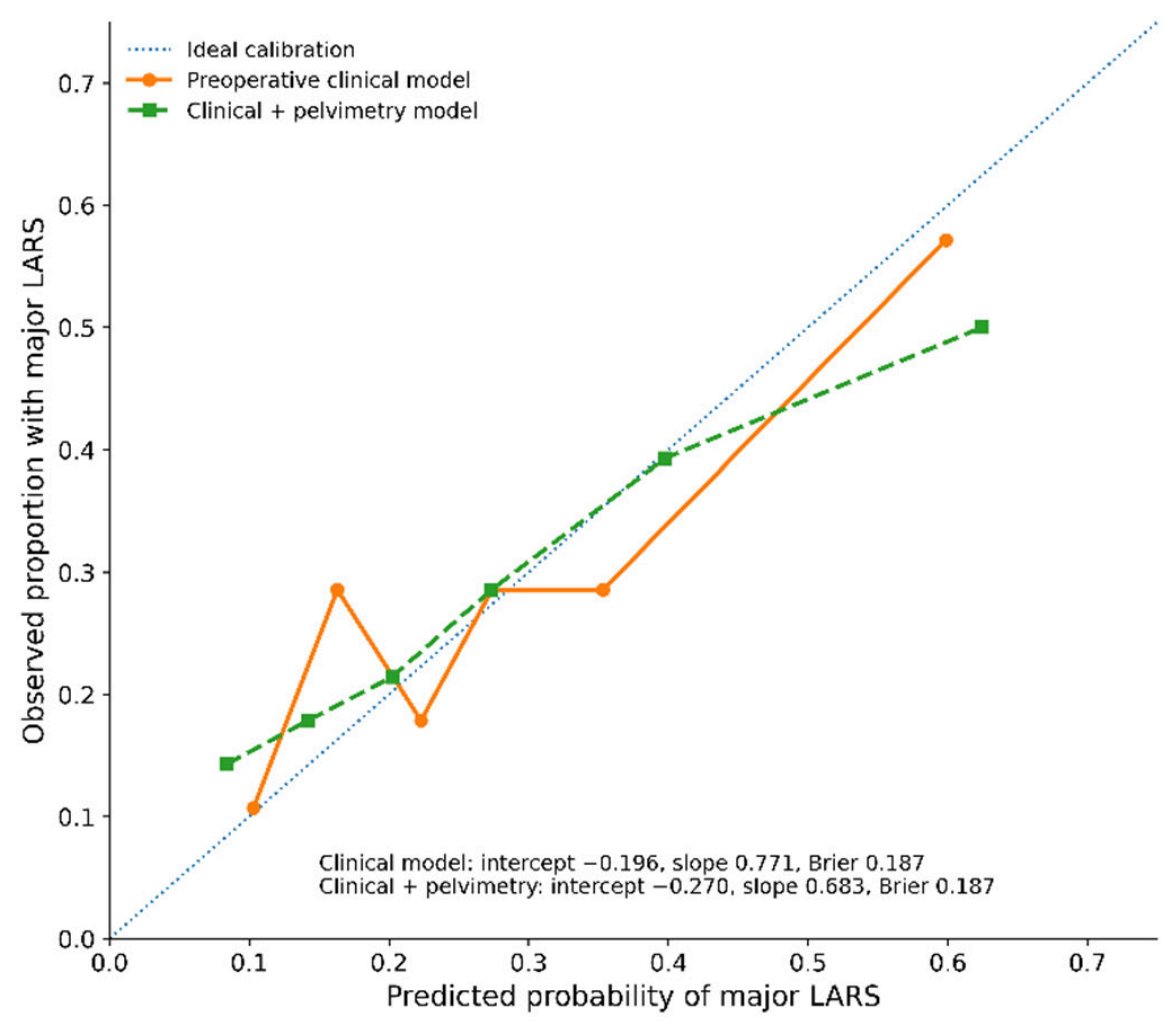
Calibration of the parsimonious preoperative clinical model and the clinical + pelvimetry model for prediction of major LARS, based on patient-level mean repeated out-of-fold predictions.

Calibration based on patient-level mean repeated out-of-fold predictions yielded an intercept of −0.196 and slope of 0.771 for the preoperative clinical model and an intercept of −0.270 and slope of 0.683 for the clinical + pelvimetry model. The Brier score was 0.187 for both models (Table 4; Figure 8).

**Figure 9:**
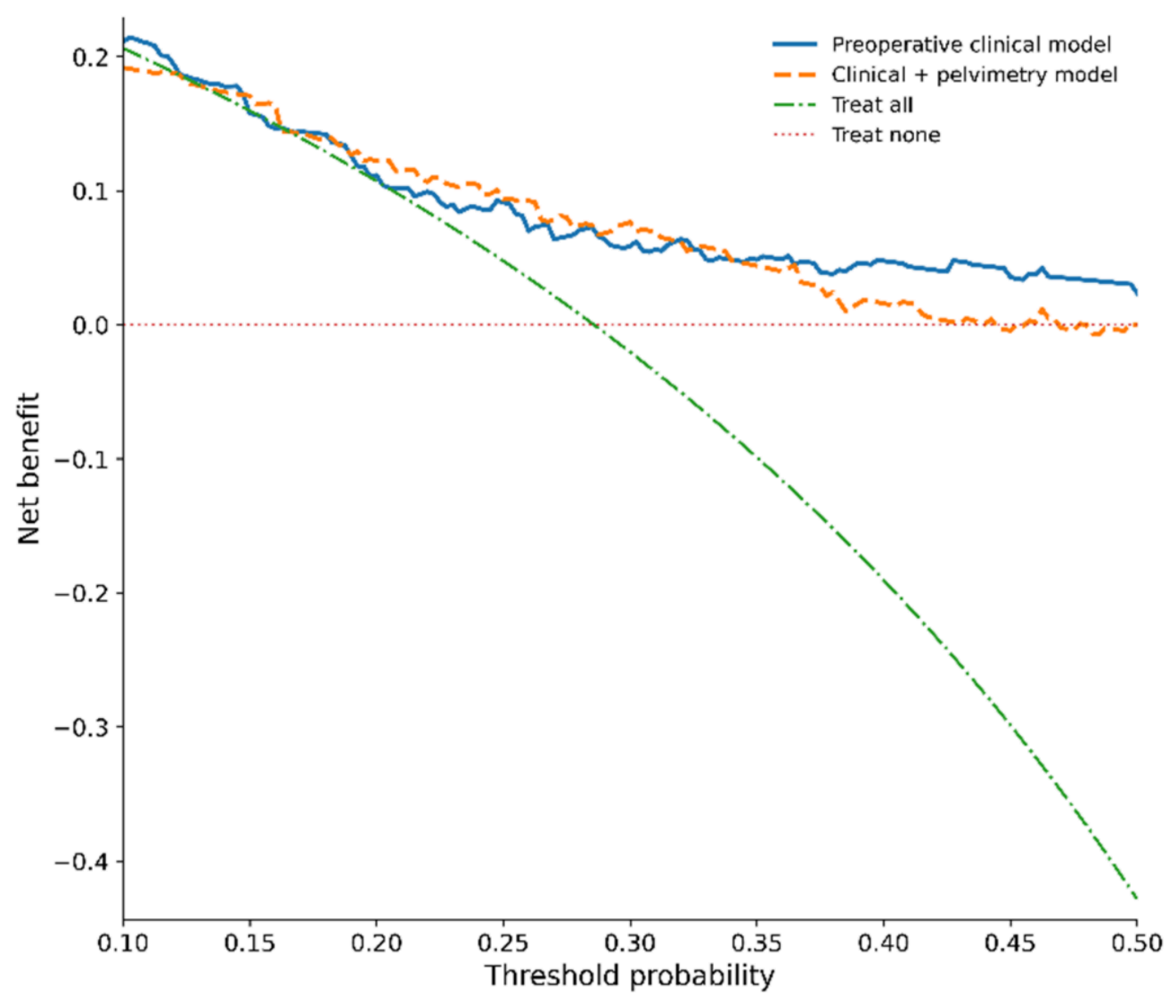
Decision-curve analysis for prediction of major LARS using the parsimonious preoperative clinical model and the clinical + pelvimetry model across threshold probabilities of 0.10–0.50.

Decision-curve analysis across threshold probabilities of 0.10–0.50 showed closely similar net-benefit profiles for the two models, with no consistent incremental advantage after addition of pelvimetry (Figure 9).

### 3.5. Adjusted Probability of Major LARS According to AP Pelvic Diameter

**Figure 10:**
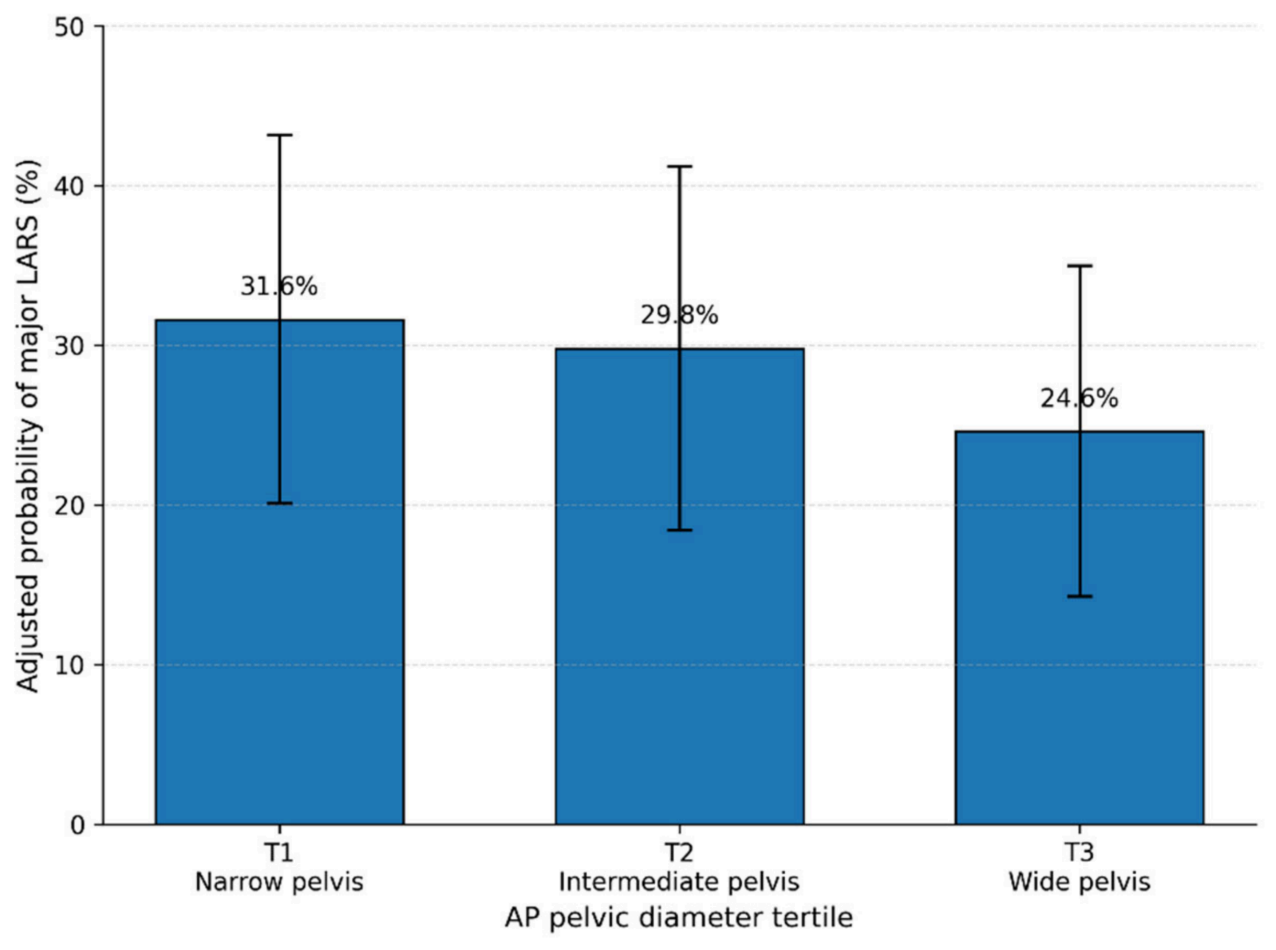
Adjusted probability of major LARS according to anteroposterior pelvic diameter tertile. Error bars represent 95% confidence intervals.

Adjusted predicted probabilities of major LARS were similar across anteroposterior pelvic diameter tertiles after adjustment for age, sex, BMI, tumor position, and diverting ileostomy. Predicted probabilities were 31.6% (95% CI 20.1–43.2%) for T1, 29.8% (95% CI 18.4–41.2%) for T2, and 24.6% (95% CI 14.3–35.0%) for T3 (Supplementary Table S8; Figure 10, Supplementary Figures S2 and S3).

## 4. Discussion

The principal finding of this study is that the addition of the three conventional MRI-derived skeletal pelvimetry measurements did not demonstrate a clear incremental improvement in prediction of major LARS beyond a parsimonious preoperative clinical reference model. The clinical model achieved a mean repeated five-fold cross-validated AUC of 0.669, compared with 0.671 after addition of pelvimetry, corresponding to a ΔAUC of +0.002 (95% CI −0.052 to 0.054), with no statistically detectable improvement in nested-model fit. In the secondary association analyses, neither anteroposterior pelvic diameter nor interspinous distance was associated with major LARS, whereas low tumor position was associated with higher odds of major LARS, although this estimate was imprecise because of the small number of low rectal tumors. Sacral curvature depth showed an exploratory inverse association in several analyses. Importantly, the confidence interval around ΔAUC does not exclude a modest incremental contribution of pelvimetry; therefore, these findings indicate no demonstrated incremental predictive benefit in this cohort rather than evidence that pelvimetry lacks predictive value.

This study represents a dedicated secondary imaging analysis of a previously reported institutional LARS cohort [25]; accordingly, the postoperative LARS outcome data themselves are not novel. Whereas the earlier study evaluated a broader range of clinical and surgical factors associated with postoperative LARS, the present investigation specifically examines the incremental predictive contribution of three conventional MRI-derived skeletal pelvimetry measurements using dedicated model-comparison, internal-validation, calibration, and sensitivity analyses.

The overall prevalence of LARS in our cohort was 43.4%, including major LARS in 28.6% of patients, consistent with previous reports after sphincter-preserving rectal cancer surgery [1,2,3,11]. Clinically relevant bowel dysfunction remains common among rectal cancer survivors and is associated with impaired quality of life [1,2,6,26,27,28,29,30,31,32]. Nevertheless, only 38.1% of patients reported symptom-directed treatment, consistent with previous observations that LARS remains underrecognized and undertreated [33,34].

Anteroposterior pelvic diameter was not associated with continuous LARS score, and the similar prevalence of major LARS across pelvic diameter tertiles provides little evidence of a clinically relevant relationship with postoperative bowel dysfunction. Sacral curvature depth showed an inverse association with major LARS across several analyses, although the magnitude and precision of the association varied. Given the evaluation of multiple correlated pelvimetry measurements, this finding should be regarded as exploratory and hypothesis-generating. One possible explanation is that greater sacral curvature may reflect slightly greater posterior pelvic capacity, potentially reducing mechanical constraints during low rectal reconstruction; however, this interpretation remains speculative. Sacral curvature depth contributed little to overall model discrimination. More broadly, these findings suggest that anatomical features associated with operative difficulty do not necessarily translate into poorer long-term bowel function.

Previous imaging studies have primarily examined pelvic dimensions as predictors of technical difficulty and perioperative outcomes rather than long-term bowel function. Narrower pelvic dimensions, reduced interspinous distance, and greater sacral depth have been associated with longer operative time, conversion, blood loss, anastomotic leakage, or technical difficulty during rectal surgery [35,36,37,38,39]. In contrast, evidence linking conventional pelvimetry directly to postoperative functional outcomes remains limited. Yuan et al. reported associations between several MRI-derived pelvic measurements and postoperative LARS, although their analysis focused on individual anatomical predictors rather than the incremental predictive value of pelvimetry beyond a clinical reference model [9]. The present findings extend this literature by evaluating whether three conventional MRI-derived skeletal pelvimetry measurements provide incremental predictive information beyond a preoperative clinical reference model; no clear incremental improvement was demonstrated in the present cohort.

Low tumor position was associated with higher odds of major LARS, consistent with previous evidence linking distal rectal tumors and low reconstruction to poorer postoperative bowel function [17,18,19,20,33,37,38,39]. Although the association persisted in Firth penalized regression, this finding should be interpreted cautiously because of the small number of patients with low rectal tumors in the present cohort. Low rectal surgery may impair reservoir capacity, anorectal sensation, and coordinated bowel emptying through more distal resection and deeper pelvic dissection. Continuous tumor distance from the anal verge was not associated with major LARS in the sensitivity analysis.

Diverting ileostomy was more frequent among patients with major LARS and showed an association in some adjusted analyses. Previous studies and meta-analyses have similarly reported an increased risk of LARS after temporary diversion [11,16,40,41,42,43,44,45]. Potential mechanisms include disuse-related changes, altered microbiota, and delayed functional adaptation after restoration of bowel continuity. However, diverting ileostomy is also preferentially used for lower anastomoses and more complex resections. Contemporary international data from the PICARR survey further indicate that decisions regarding protective diversion after anterior rectal resection are influenced by factors including low anastomotic level, neoadjuvant treatment, and technical considerations [46]. It should therefore be regarded both as a potential contributor to postoperative dysfunction and as a marker of a more demanding surgical pathway. Other surgical and postoperative events may similarly contribute to long-term functional outcomes. Anastomotic leakage and pelvic septic complications represent potentially relevant factors in LARS development, although they were not included in the preoperative prediction model because they are not available at the intended prediction time point; contemporary SICCR survey data further highlight the heterogeneity of their prevention and management in colorectal practice [47].

The parsimonious preoperative clinical model demonstrated modest discrimination, with a mean repeated five-fold cross-validated AUC of 0.669, consistent with contemporary LARS models that generally report AUC values between approximately 0.65 and 0.85 [20,21,39,41,48,49,50,51]. The addition of the three conventional MRI pelvimetry measurements yielded an AUC of 0.671 (ΔAUC = +0.002, 95% CI −0.052 to 0.054) and did not produce a statistically detectable improvement in nested-model fit. The confidence interval around ΔAUC was compatible with both a small decrease and a modest increase in discrimination, emphasizing that the present analysis cannot exclude a smaller incremental contribution from pelvimetry. Calibration based on patient-level mean repeated out-of-fold predictions yielded slopes of 0.771 for the clinical model and 0.683 for the clinical + pelvimetry model, with identical Brier scores of 0.187. Calibration slopes below 1 indicate residual overfitting, particularly in the extended model, despite the more parsimonious specification and internal validation. Decision-curve analysis showed closely similar net-benefit profiles for the two models, with no consistent incremental advantage after addition of pelvimetry. These findings therefore support the interpretation that no clear incremental predictive benefit was demonstrated in this cohort, while underscoring the need for larger datasets and external validation.

The absence of a clear incremental predictive benefit from conventional pelvimetry in the present cohort is biologically plausible because LARS is multifactorial, reflecting interactions among loss of the native rectal reservoir, autonomic denervation, altered colonic motility, sphincter and pelvic floor dysfunction, impaired anorectal sensation, radiotherapy-related injury, and postoperative adaptation [11,34,50]. Emerging evidence also suggests roles for changes in the intestinal microbiome and neuromuscular coordination [51,52]. Conventional skeletal measurements do not directly capture nerve preservation, neorectal compliance, pelvic floor function, or anorectal physiology. Importantly, the absence of a clear incremental predictive benefit was unlikely to reflect poor measurement reproducibility, as interobserver agreement was excellent for all three measurements (ICC > 0.98). Future prediction models will likely require integration of clinical, surgical, functional, and biological factors rather than reliance on skeletal morphology alone.

This study has several limitations. First, its retrospective single-center design may limit generalizability. Second, the relatively small number of major-LARS events reduced statistical precision and increased the risk of model overfitting; although the primary prediction model was deliberately kept parsimonious and internally validated, the available data remain insufficient to exclude modest incremental predictive effects of pelvimetry. Third, only a small proportion of patients had low rectal tumors, resulting in substantial uncertainty and wide confidence intervals around the corresponding effect estimate; although the association persisted in Firth penalized regression, the limited subgroup size precludes a precise estimate of its magnitude. Fourth, postoperative functional assessment was performed at heterogeneous time points, and major LARS was more frequent among patients assessed at earlier intervals after surgery or restoration of bowel continuity. Because bowel function may change through postoperative adaptation, this temporal heterogeneity may have influenced both prevalence estimates and predictor associations. However, the principal pelvimetry findings remained materially unchanged when analyses were restricted to patients assessed ≥12 months after surgery or restoration of bowel continuity. Postoperative bowel function was assessed using a Romanian-language translation of the LARS questionnaire that has not undergone formal linguistic or psychometric validation, and formal inter-interviewer agreement was not evaluated. Although both interviewers received standardized training and administered the questionnaire using predefined questions and response categories, potential measurement error or outcome misclassification related to translation or interviewer administration cannot be excluded. In addition, standardized preoperative LARS assessments were not available, precluding adjustment for baseline bowel function and limiting the ability to distinguish postoperative symptoms from pre-existing bowel dysfunction. Selection bias is also possible. Patients excluded because of incomplete pelvimetry differed from those included in tumor position, neoadjuvant radiotherapy, and diverting ileostomy. In addition, the functional cohort included only patients who survived to assessment, achieved bowel continuity, and completed the questionnaire, introducing potential survivorship and response bias. Patients with more complicated postoperative trajectories may therefore have been underrepresented, although the overall direction of this bias cannot be determined from the available data.

Despite these limitations, this study provides a comprehensive evaluation of three conventional MRI-derived skeletal pelvimetry measurements using complementary association and prediction analyses, including repeated cross-validation, calibration, and decision-curve analysis. Across these approaches, the addition of pelvimetry did not demonstrate a clear incremental predictive benefit beyond the parsimonious preoperative clinical reference model. Confirmation in larger, prospectively characterized cohorts with standardized functional assessment and external validation is warranted.

## 5. Conclusions

In this selected retrospective cohort, the three conventional MRI-derived skeletal pelvimetry measurements evaluated did not demonstrate a clear incremental improvement in prediction of major LARS beyond the parsimonious preoperative clinical reference model. Future prediction models should integrate clinical, anatomical, surgical, functional, and biological information to improve individualized risk assessment and guide patient counseling.

## Figures and Tables

**Table 1:** Baseline characteristics according to LARS severity.

Variable	Overall	No LARS	Minor LARS	Major LARS	*p*-Value
Age, years	67 (58–71.3)	68 (60–72)	67 (64–73)	64 (54.5–69)	0.025
Male sex, n (%)	91 (54.2)	47 (49.5)	16 (64.0)	28 (58.3)	0.341
BMI, kg/m^2^	27.7 (24.7–30.1)	27.4 (24.0–29.7)	27.9 (25.1–30.4)	28.4 (26.0–30.5)	0.482
Diabetes mellitus, n (%)	27 (16.1)	16 (16.8)	6 (24.0)	5 (10.4)	0.310
Smoking history, n (%)	76 (45.2)	36 (37.9)	14 (56.0)	26 (54.2)	0.092
Tumor position, n (%)					<0.001
− Upper rectum	69 (41.1)	45 (47.4)	11 (44.0)	13 (27.1)	
− Middle rectum	88 (52.4)	50 (52.6)	11 (44.0)	27 (56.2)	
− Lower rectum	11 (6.5)	0 (0.0)	3 (12.0)	8 (16.7)	
Neoadjuvant radiotherapy, n (%)	102 (60.7)	51 (53.7)	15 (60.0)	36 (75.0)	0.048
Chemotherapy (any), n (%)	126 (75.0)	65 (68.4)	21 (84.0)	40 (83.3)	0.080
Diverting ileostomy, n (%)	111 (66.1)	54 (56.8)	17 (68.0)	40 (83.3)	0.007
Tumor distance from the anal verge, mm	85 (70–107.5)	88.5 (70–110); n = 68	87 (70–100); n = 19	82.5 (71.5–110); n = 44	0.815
Surgical approach, n (%)					0.656
Open	149 (88.7)	86 (90.5)	22 (88.0)	41 (85.4)	
Robotic	19 (11.3)	9 (9.5)	3 (12.0)	7 (14.6)	
Mesorectal excision, n (%)					0.281
TME	126 (75.0)	67 (70.5)	21 (84.0)	38 (79.2)	
PME	42 (25.0)	28 (29.5)	4 (16.0)	10 (20.8)	
Anastomotic configuration, n (%)					0.763
End-to-end	117 (69.6)	64 (67.4)	18 (72.0)	35 (72.9)	
Side-to-end	51 (30.4)	31 (32.6)	7 (28.0)	13 (27.1)	
Anastomotic technique, n (%)					0.085
Stapled	162 (96.4)	94 (98.9)	24 (96.0)	44 (91.7)	
Hand-sewn	6 (3.6)	1 (1.1)	1 (4.0)	4 (8.3)	
Anastomotic leakage/pelvic sepsis, n (%)	15 (8.9)	8 (8.4)	3 (12.0)	4 (8.3)	0.843
MRI pelvimetry: AP diameter, mm	112.5 (104–121)	113 (104–121)	112 (101–118)	113 (104–119.8)	0.777
MRI pelvimetry: interspinous distance, mm	99 (90–108.3)	99 (90–110.5)	97 (88–106)	98 (91.4–108)	0.711
MRI pelvimetry: sacral curvature depth, mm	32 (25–38.3)	33 (26–39.5)	32 (24.5–41)	28 (24–36.3)	0.178
Time to ileostomy reversal, months	1.4 (0–2.4)	1.6 (1.1–2.9)	1.7 (0.9–2.4)	1.3 (0–2.0)	0.301
Time to LARS assessment, months	31.0 (14.6–66.7)	36.1 (18.3–69.1)	34.9 (16.8–73.3)	20.7 (12.5–46.7)	0.048

**Note**: Continuous variables are presented as median (interquartile range), and categorical variables as number (%). The overall column is descriptive and was not included in hypothesis testing. Continuous variables were compared using the Kruskal–Wallis test, and categorical variables using the χ^2^ test or Fisher’s exact test, as appropriate. Tumor-distance analyses included 131 patients with valid measurements; values recorded as 0 mm were treated as missing because they represented unavailable measurements. Time to ileostomy reversal was calculated among patients with a diverting ileostomy and documented reversal timing. Time to LARS assessment was available for 167 patients.

**Table 2:** Postoperative LARS outcomes according to anteroposterior pelvic diameter tertile.

AP Pelvic Diameter Tertile	LARS Score, Median (IQR)	Major LARS, n (%)
T1, narrow pelvis (n = 57)	18 (7–30)	16 (28.1)
T2, intermediate pelvis (n = 55)	18 (11–30.5)	17 (30.9)
T3, wide pelvis (n = 56)	16 (8.5–32)	15 (26.8)
*p*-value	0.622	0.886

**Note**: LARS, low anterior resection syndrome; IQR, interquartile range. LARS scores were compared using the Kruskal–Wallis test, and major LARS prevalence using the χ^2^ test.

**Table 3:** Adjusted associations of clinical and conventional pelvimetry variables with major LARS.

Predictor	Adjusted OR	95% CI	*p*-Value
AP pelvic diameter (per 10-mm increase)	1.04	0.77–1.41	0.795
Interspinous distance (per 10-mm increase)	0.97	0.70–1.34	0.840
Sacral curvature depth (per 10-mm increase)	0.65	0.44–0.98	0.042
Middle vs. upper rectal tumor	1.33	0.56–3.14	0.513
Low vs. upper rectal tumor	9.51	2.00–45.15	**0.005**
Diverting ileostomy (yes vs. no)	2.58	0.98–6.77	0.054

**Note**: Adjusted odds ratios were estimated using multivariable logistic regression. The model included AP pelvic diameter, interspinous distance, sacral curvature depth, categorical tumor position, and diverting ileostomy. Upper rectal tumor position served as the reference category. Outcome: major LARS versus no/minor LARS. OR, odds ratio; CI, confidence interval.

**Table 4:** Predictive performance of the parsimonious preoperative clinical reference model and clinical + MRI pelvimetry model for major LARS.

Performance Measure	Preoperative Clinical Model	Clinical + Pelvimetry Model
Model parameters, *n*	4	7
Mean repeated 5-fold CV AUC	0.669	0.671
ΔAUC vs. clinical model	Reference	+0.002
95% CI for ΔAUC	—	−0.052 to 0.054
Calibration intercept	−0.196	−0.270
Calibration slope	0.771	0.683
Brier score	0.187	0.187
Likelihood-ratio χ^2^	—	5.79
Degrees of freedom	—	3
Likelihood-ratio *p*-value	—	0.122

**Note**: The preoperative clinical reference model included age, categorical tumor position, and neoadjuvant radiotherapy; categorical tumor position was represented by two indicator variables. The clinical + pelvimetry model additionally included AP pelvic diameter, interspinous distance, and sacral curvature depth. AUC values represent mean out-of-fold AUCs from 100 repetitions of stratified five-fold cross-validation using identical resampling partitions for both models. The 95% CI for ΔAUC was obtained using 10,000 paired patient-level bootstrap resamples of patient-level mean repeated out-of-fold predictions. Calibration intercepts, slopes, and Brier scores were calculated from these internally validated predictions. The nested maximum-likelihood model comparison yielded LR χ^2^ = 5.79, df = 3, *p* = 0.122. AUC, area under the receiver operating characteristic curve; CI, confidence interval; CV, cross-validation; LR, likelihood-ratio; AP, anteroposterior; LARS, low anterior resection syndrome.

## Data Availability

The data supporting the findings of this study are available from the corresponding author upon reasonable request.

## Supplementary Materials

The following supporting information can be downloaded at https://www.mdpi.com/article/10.3390/jcm15187145/s1, Table S1: Baseline characteristics of patients included in the complete-case pelvimetry cohort and patients excluded because of incomplete pelvimetry data; Table S2: Correlation matrix between pelvic anatomical parameters, clinical variables, and postoperative functional outcomes; Table S3: Interobserver reproducibility of MRI pelvimetry measurements; Table S4: Firth penalized logistic regression sensitivity analysis for major LARS in the full pelvimetry cohort; Table S5: Sensitivity multivariable analysis additionally adjusted for time to LARS assessment; Table S6: Postoperative LARS outcomes according to interval to functional assessment; Table S7: Sensitivity multivariable analysis restricted to patients assessed ≥12 months after surgery or restoration of bowel continuity; Table S8: Multivariable logistic regression for major LARS using AP pelvic diameter tertiles and adjusted predicted prevalence estimates; Table S9: Specification and internal validation of the primary prediction models for major LARS; Figure S1: Postoperative LARS outcomes according to interval to functional assessment; Figure S2: Adjusted odds ratios for major LARS according to AP pelvic diameter tertile; Figure S3: Adjusted probability of major LARS across MRI-measured anteroposterior pelvic diameter modeled using restricted cubic splines.

## Abbreviations

The following abbreviations are used in this manuscript:

AP	anteroposterior
AUC	area under the receiver operating characteristic curve
BMI	body mass index
CI	confidence interval
CT	computed tomography
CV	cross-validation
df	degrees of freedom
ICC	intraclass correlation coefficient
IQR	interquartile range
LARS	low anterior resection syndrome
MRI	magnetic resonance imaging
OR	odds ratio
ROC	receiver operating characteristic
SE	standard error
STROBE	Strengthening the Reporting of Observational Studies in Epidemiology
TME	total mesorectal excision
