# Supplementary material for: Incremental Predictive Value of Conventional MRI Pelvimetry for Major Low Anterior Resection Syndrome After Rectal Cancer Surgery
Source: J Clin Med. 2026 Sep 15;15(18):7145. doi: 10.3390/jcm15187145 (PMC13607746; doi:10.3390/jcm15187145)
Supplement: Supplementary file 1 [file jcm-15-07145-s001.zip › jcm-4493275-supplementary.pdf]

Supplementary Table S1. Baseline characteristics of patients included in the complete-case pelvimetry cohort and patients excluded because of incomplete pelvimetry data

Variable	Included (n = 168)	Excluded (n = 14)	p value
Age, years	64.6 ± 9.9	67.3 ± 10.9	0.378
Male sex, n (%)	91 (54.2)	8 (57.1)	1.000
BMI, kg/m²	27.6 ± 4.4	28.8 ± 6.4	0.509
Tumor position, upper/middle/lower, n	69 / 88 / 11	10 / 2 / 2	0.022
Neoadjuvant radiotherapy, n (%)	102 (60.7)	4 (28.6)	0.024
Chemotherapy, n (%)	126 (75.0)	10 (71.4)	0.754
Diverting ileostomy, n (%)	111 (66.1)	5 (35.7)	0.039

Note: Data are presented as mean ± standard deviation or n (%). Continuous variables were compared using Welch's *t*-test. Binary categorical variables were compared using Fisher's exact test, and tumor position was compared using the χ^2 test.

Supplementary Table S2. Correlation matrix between pelvic anatomical parameters, clinical variables, and postoperative functional outcomes

Variable	LARS score	Major LARS	AP diameter	Interspinous distance	Sacral curvature depth	Distance from anal verge
**LARS score**	1.00	0.76	0.03	0.01	−0.20	−0.01
**Major LARS**	0.76	1.00	0.02	0.00	−0.14	−0.02
**AP diameter**	0.03	0.02	1.00	0.19	−0.05	−0.04
**Interspinous distance**	0.01	0.00	0.19	1.00	−0.17	−0.01
**Sacral curvature depth**	−0.20	−0.14	−0.05	−0.17	1.00	−0.08
**Distance from anal verge**	−0.01	−0.02	−0.04	−0.01	−0.08	1.00

Note: Spearman correlation coefficients (ρ) are presented. Major LARS was coded as a binary variable (major LARS versus no/minor LARS). Correlations involving distance from the anal verge were calculated using the 131 patients with valid positive measurements; recorded values of 0 mm were treated as missing. Positive coefficients indicate direct associations, whereas negative coefficients indicate inverse associations. AP, anteroposterior; LARS, low anterior resection syndrome.

Supplementary Table S3. Interobserver reproducibility of MRI pelvimetry measurements

Measurement	n	ICC(2,1)	95% CI	Mean difference (R1−R0), mm	95% limits of agreement, mm
AP pelvic diameter	168	0.992	0.990–0.994	+0.19	−2.83 to +3.21
Interspinous distance	168	0.990	0.988–0.992	0.00	−3.27 to +3.27
Sacral curvature depth	168	0.985	0.981–0.988	+0.11	−3.01 to +3.22

Note: ICC, intraclass correlation coefficient; CI, confidence interval. ICCs were estimated using a two-way random-effects, absolute-agreement, single-measurement model [ICC(2,1)]. Mean differences were calculated as second-reader minus primary-reader measurements.

Supplementary Table S4. Firth penalized logistic regression sensitivity analysis for major LARS in the full pelvimetry cohort

Predictor	Penalized OR	95% profile-likelihood CI	p value
AP pelvic diameter, per 10 mm increase	1.04	0.77–1.40	0.803
Interspinous distance, per 10 mm increase	0.97	0.70–1.34	0.852
Sacral curvature depth, per 10 mm increase	0.67	0.45–1.00	0.052
Middle vs upper rectal tumor	1.31	0.57–3.07	0.528
Low vs upper rectal tumor	7.96	1.97–38.95	**0.003**
Diverting ileostomy, yes vs no	2.44	0.99–6.48	0.054

Note: The outcome was major LARS versus no/minor LARS. The model included AP pelvic diameter, interspinous distance, sacral curvature depth, categorical tumor position, and diverting ileostomy. Upper rectal tumor position was the reference category. Odds ratios were estimated using Firth penalized logistic regression. Confidence intervals are profile-likelihood intervals, and p values were obtained using penalized likelihood-ratio tests. AP, anteroposterior; OR, odds ratio; CI, confidence interval; LARS, low anterior resection syndrome.

Supplementary Table S5. Sensitivity multivariable analysis additionally adjusted for time to LARS assessment

Predictor	Adjusted OR	95% CI	p value
AP pelvic diameter, per 10 mm increase	1.05	0.77–1.44	0.751
Interspinous distance, per 10 mm increase	0.97	0.70–1.35	0.853
Sacral curvature depth, per 10 mm increase	0.63	0.41–0.96	0.031
Middle vs upper rectal tumor	1.18	0.49–2.83	0.717
Low vs upper rectal tumor	10.04	1.92–52.56	0.006
Diverting ileostomy, yes vs no	2.73	1.02–7.34	0.046
Time to LARS assessment, per month	0.986	0.974–0.998	0.023

Note: The outcome was major LARS versus no/minor LARS. The model included AP pelvic diameter, interspinous distance, sacral curvature depth, categorical tumor position, diverting ileostomy, and time to LARS assessment. Upper rectal tumor position was the reference category. The analysis included 167 patients with complete data for all model variables. OR, odds ratio; CI, confidence interval; AP, anteroposterior; LARS, low anterior resection syndrome.

Supplementary Table S6. Postoperative LARS outcomes according to interval to functional assessment

Interval to LARS assessment	n	Major LARS, n (%)	LARS score, median
<12 months	28	12 (42.9)	26
12–24 months	38	15 (39.5)	19
24–60 months	48	13 (27.1)	18
≥60 months	53	8 (15.1)	16

Note: Assessment interval was calculated from surgery in patients without a diverting ileostomy and from ileostomy closure in patients who underwent diversion. Assessment-time data were available for 167 patients.

Supplementary Table S7. Sensitivity multivariable analysis restricted to patients assessed ≥12 months after surgery or restoration of bowel continuity

Predictor	Adjusted OR	95% CI	*p* value
AP pelvic diameter (per 10-mm increase)	**1.11**	**0.79–1.55**	0.561
Interspinous distance (per 10-mm increase)	**1.02**	**0.71–1.45**	0.927
Sacral curvature depth (per 10-mm increase)	**0.68**	**0.43–1.08**	0.101
Middle vs upper rectal tumor	1.52	0.57–4.04	0.400
Low vs upper rectal tumor	3.20	0.39–26.43	0.281
Diverting ileostomy (yes vs no)	1.88	0.64–5.52	0.253

Note: The sensitivity analysis was restricted to patients whose functional assessment occurred ≥12 months after surgery or restoration of bowel continuity. Of 139 patients meeting the timing criterion, 138 had complete data for all variables included in the multivariable model. The outcome was major LARS versus no/minor LARS. The model included AP pelvic diameter, interspinous distance, sacral curvature depth, categorical tumor position, and diverting ileostomy. Upper rectal tumor position was the reference category. Joint addition of the three pelvimetry measurements did not improve model fit (likelihood-ratio χ^2 = **3.65**, df = 3; *p* = **0.302**). OR, odds ratio; CI, confidence interval; AP, anteroposterior; LARS, low anterior resection syndrome.

Supplementary Table S8. Multivariable logistic regression for major LARS using AP pelvic diameter tertiles and adjusted predicted prevalence estimates

Part A. Multivariable logistic regression

Predictor	Adjusted OR	95% CI	p value
AP tertile 2 vs tertile 1	0.90	0.36–2.28	0.826
AP tertile 3 vs tertile 1	0.66	0.25–1.71	0.391
Age, per year	0.94	0.91–0.98	0.004
Male sex, vs female	1.01	0.46–2.23	0.971
BMI, per kg/m²	1.07	0.98–1.17	0.155
Middle vs upper rectal tumor	0.94	0.38–2.32	0.888
Low vs upper rectal tumor	7.46	1.56–35.67	0.012
Diverting ileostomy, yes vs no	3.41	1.27–9.20	0.015

Part B. Adjusted predicted prevalence of major LARS

AP pelvic diameter tertile	Adjusted prevalence	95% CI
T1, narrow pelvis	31.6%	20.1%–43.2%
T2, intermediate pelvis	29.8%	18.4%–41.2%
T3, wide pelvis	24.6%	14.3%–35.0%

Note: The outcome was major LARS versus no/minor LARS. The multivariable model included AP pelvic diameter tertile, age, sex, BMI, categorical tumor position, and diverting ileostomy. T1 and upper rectal tumor position were the reference categories. Adjusted prevalence estimates were calculated as average marginal predicted probabilities, with 95% confidence intervals estimated using the delta method. The analysis included 168 patients and 48 major LARS events. AP, anteroposterior; BMI, body mass index; OR, odds ratio; CI, confidence interval; LARS, low anterior resection syndrome.

Supplementary Table S9. Specification and internal validation of the primary prediction models for major LARS

Component	Preoperative clinical model	Clinical + pelvimetry model
Age	Included	Included
Tumor position	Included (2 indicator variables)	Included (2 indicator variables)
Neoadjuvant radiotherapy	Included	Included
AP pelvic diameter	—	Included
Interspinous distance	—	Included
Sacral curvature depth	—	Included
Total model parameters	4	7
Internal validation	Stratified 5-fold CV repeated 100 times	Stratified 5-fold CV repeated 100 times
Resampling partitions	Identical paired partitions	Identical paired partitions
Mean CV AUC	0.669	0.671
Calibration intercept	−0.196	−0.270
Calibration slope	0.771	0.683
Brier score	0.187	0.187

Note: The primary prediction models included only variables available at the intended preoperative prediction time point. Stratified five-fold cross-validation was repeated 100 times, preserving the proportion of major LARS events within each fold and using identical resampling partitions for the paired model comparison. For each repetition, predictions from the five held-out folds were combined to obtain out-of-fold predictions. Patient-level mean repeated out-of-fold predicted probabilities were used for calibration assessment and calculation of Brier scores. The 95% confidence interval for the paired difference in AUC was estimated using 10,000 paired patient-level bootstrap resamples of these predictions. CV, cross-validation; AUC, area under the receiver operating characteristic curve; AP, anteroposterior; LARS, low anterior resection syndrome.

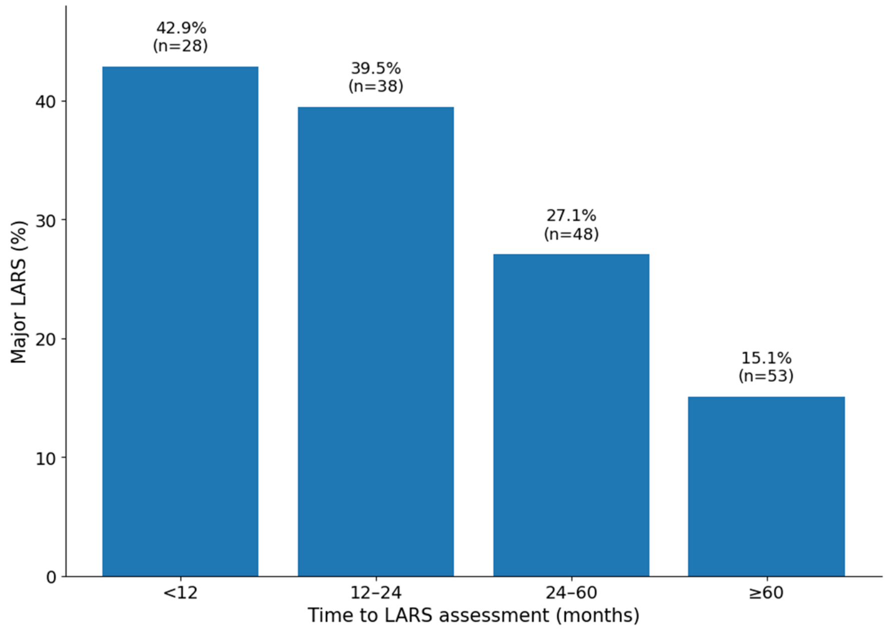

Supplementary Figure S1. Postoperative LARS outcomes according to interval to functional assessment

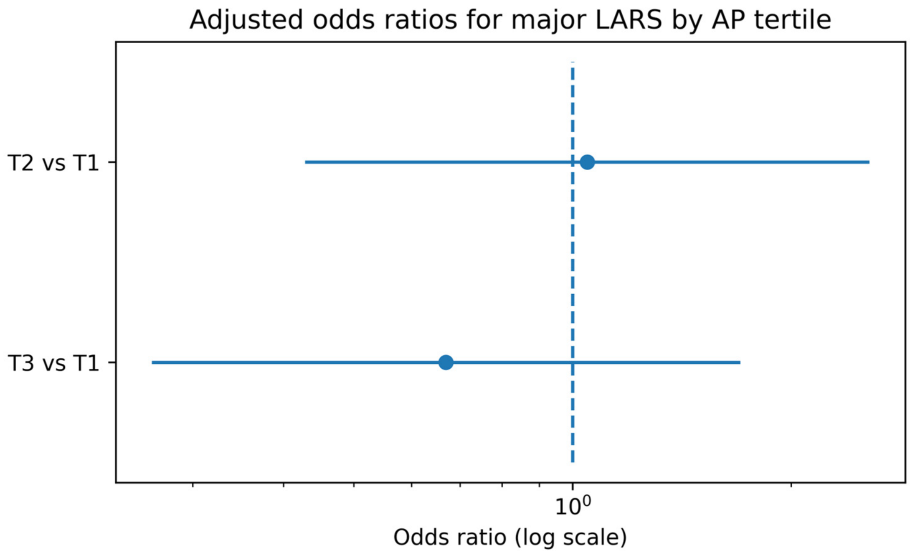

Supplementary Figure S2. Adjusted odds ratios for major LARS according to AP pelvic diameter tertile. T1 was the reference category. Estimates were derived from the multivariable model presented in Supplementary Table S8.

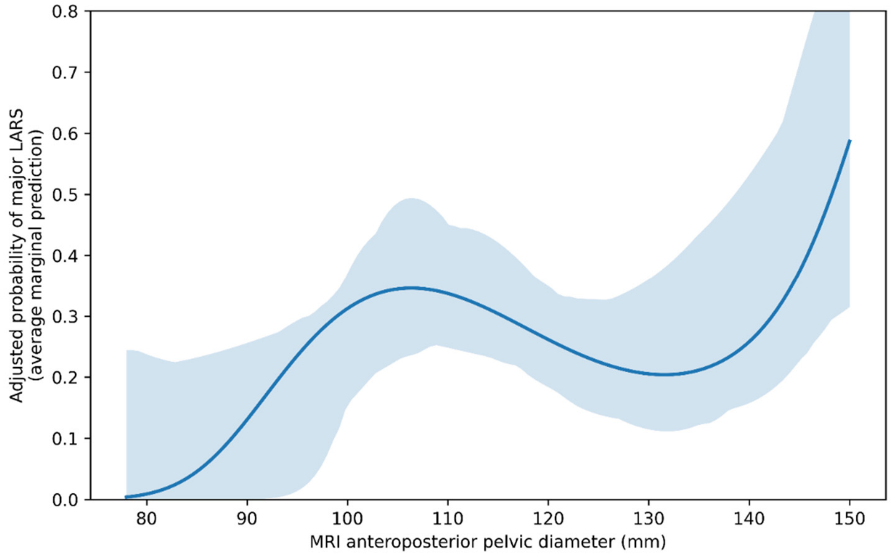

Supplementary Figure S3. Adjusted probability of major LARS across MRI-measured anteroposterior pelvic diameter modeled using restricted cubic splines. The solid line represents the estimated probability, and the shaded area represents the 95% confidence interval. Estimates were adjusted for the clinical covariates included in the model.
